# The Great Mimicker: Extragonadal Endometriosis Presenting as an Appendiceal Mass and Acute Appendicitis

**DOI:** 10.7759/cureus.88718

**Published:** 2025-07-25

**Authors:** Jinyoung Ha, Fatima Hamid, Jane Ahn, Nicole Afuape, Erum Azhar

**Affiliations:** 1 Obstetrics and Gynecology, A.T. Still University College of Osteopathic Medicine in Arizona, Mesa, USA; 2 Obstetrics and Gynecology, Dignity Health East Valley, Obstetrics and Gynecology Residency Program, Gilbert, USA; 3 Obstetrics and Gynecology, Creighton University School of Medicine, Phoenix, USA

**Keywords:** acute abdomen, appendectomy, appendiceal endometriosis, appendiceal mass, appendicitis, endometriosis, extragonadal endometriosis, right lower quadrant abdominal pain

## Abstract

A 45-year-old female presented to the Emergency Department with acute right lower quadrant (RLQ) pain. Her past medical history was significant for heavy menstrual bleeding and moderate dysmenorrhea, though a diagnosis of endometriosis had never been established previously. Initial work-up revealed anemia and leucocytosis. A CT of the abdomen and pelvis showed an enlarged structure in RLQ along the medial border of the cecum, suspicious for acute appendicitis with a retained rupture. CA19-9, CA-125, and CEA were sent, of which only CA-125 was elevated at 92.7 U/mL (reference range: 0-35 U/mL). She was admitted to the hospital for complicated appendicitis and was started on IV piperacillin-tazobactam. A repeat CT scan to monitor response to antibiotics showed acute appendicitis along with phlegmon with a small amount of free fluid, and an enlarged heterogeneous uterus with thickened endometrium, uterine fibroids, and a left ovarian cyst. A CT-guided drainage by Interventional Radiology (IR) was deferred due to concerns for a suspected malignant appendiceal mass, and she was treated with 14 days of antibiotics in the setting of complicated appendicitis.

An appendiceal biopsy via colonoscopy after discharge revealed a serrated adenoma. The patient underwent a laparoscopic appendectomy and a right hemicolectomy due to concern for appendiceal malignancy. The final histopathology showed a 2.8 cm endometriotic mass involving the wall of the appendix and surrounding tissue, one diverticulum of the colon, and was negative for dysplasia and malignancy. She had a further gynecologic workup with an in-office hysteroscopy, endometrial biopsy, and a pelvic MRI. The pathology showed no evidence of atypical hyperplasia or malignancy. The MRI revealed numerous uterine leiomyomas, adenomyosis, and a left adnexal endometrioma measuring 3.6 x 3.2 cm. She underwent a subsequent robotic-assisted total hysterectomy, bilateral salpingectomy, left oophorectomy, right ovarian cystectomy, and extensive adhesiolysis/enterolysis with intraoperative colorectal surgery consultation. Her post-operative course was uncomplicated. This report highlights the importance of considering extragonadal endometriosis in women of reproductive age as a differential diagnosis for RLQ pain and mass. It also underscores the importance of investigating the preoperative findings of appendiceal masses in women of childbearing age with appendiceal endometriosis presenting as an acute abdomen.

## Introduction

Endometriosis is characterized by the presence of endometrial glands and stroma in ectopic locations outside the uterus. [1,2]. It affects about 10-15% of women and 35-50% of women of reproductive age with pelvic pain and infertility; however, the average time to diagnosis is approximately 10 years [3,4]. Endometriosis is most commonly found in the pelvis, typically involving the peritoneum, ovaries, and uterosacral ligaments [5,6]. Endometriomas, a common manifestation of endometriosis, are endometrial cysts characterized by their viscous, dark brown, tar-like fluid. These cysts and endometrial deposits can also be found in extragonadal, extra-pelvic areas, and, in more severe cases, are productive of fibrotic change, which can result in thickened scarring and adherence to surrounding tissues.

Clinical manifestations of endometriosis can be mistaken for other pathological masses such as malignancy, lipomas, hernias, and abscesses until further evaluation is pursued surgically [7]. One entity linked to extragonadal endometriosis is endometriosis of the appendix, considered a rare presentation in the general population [8]. Appendiceal endometriosis has a prevalence of 0.4-1% among the general population [9]. Among patients with a diagnosis of endometriosis, 8-12% have bowel involvement, with the rectum and sigmoid colon making up 90% of these cases [10]. The manifestations of endometriosis in the gastrointestinal (GI) tract can vary. Patients can be asymptomatic. Others may exhibit symptoms overlapping with those commonly seen in irritable bowel syndrome, such as painful bowel movements and bleeding.

Hale et al. reported that appendiceal masses, typically inflammatory phlegmon, are present in 2-6% of appendicitis cases [11]. Numerous other etiologies, such as primary malignancy, metastatic disease, and complex pathologies, can necessitate appendectomy. Notably, appendiceal endometriosis is identified in less than 1% of females during post-appendectomy histological examination. It is challenging to diagnose appendiceal endometrioma preoperatively due to a lack of distinguishing radiological features [12]. Additional data is needed to confirm if a patient presenting with appendicitis may be at an elevated risk of an underlying pathology.

To the best of our knowledge, there are a limited number of cases reported of endometriosis that mimic acute appendicitis and appendiceal mass in gynecological literature. Our case is unique since the patient, without prior evaluation for endometriosis, exhibited all the signs and symptoms of acute appendicitis. Hence, it posed the dilemma of differentiating the mass from a common diagnosis, appendiceal malignancy, or rarer appendiceal endometriosis in the differential diagnosis. This report highlights the importance of considering the rare differential diagnosis of extragonadal endometriosis in women of reproductive age, among emergency room physicians, gynecologists, and general surgeons. This may benefit patients presenting with atypical symptoms, irrespective of a prior endometriosis history, as appendiceal endometriosis can manifest as an isolated finding.

This article was previously presented as a meeting abstract at the 2025 Arizona Academic Family Medicine Innovation Conference on April 4, 2025, as well as the Arizona Medical Association Meeting: Advocacy Summit on April 5, 2025.

## Case presentation

A 45-year-old female presented to the Emergency Department with acute, persistent, and worsening right lower quadrant (RLQ) abdominal pain initially associated with her menses. The patient characterized the pain as sharp and unremitting, and rated its intensity as moderate to severe. She reported no associated fever, nausea, or vomiting. The patient had a history of moderate dysmenorrhea; however, there had been no prior evaluation for endometriosis. Her dysmenorrhea had notably worsened to severe over the preceding menses. She reported a history of heavy menstrual bleeding lasting seven days, with three days of heavier bleeding. Her past surgical history was significant for vaginal rejuvenation and breast augmentation. On physical examination, the patient was normotensive with a blood pressure of 115/64 mmHg and a pulse of 91. Abdominal examination revealed severe RLQ pain and pelvic tenderness with guarding. As the patient presented with acute worsening and persistence of RLQ abdominal pain initially associated with her menstrual cycles, the differentials were broad. It included both non-gynecological and gynecological etiologies for acute abdomen, such as ovarian torsion, tubo-ovarian abscess, hemorrhagic ovarian cyst, pelvic congestion syndrome, seroma, lipoma, incarcerated inguinal hernia, enlarged lymph nodes, abdominal/incisional wall endometrioma, and malignancy.

**Figure 1:**
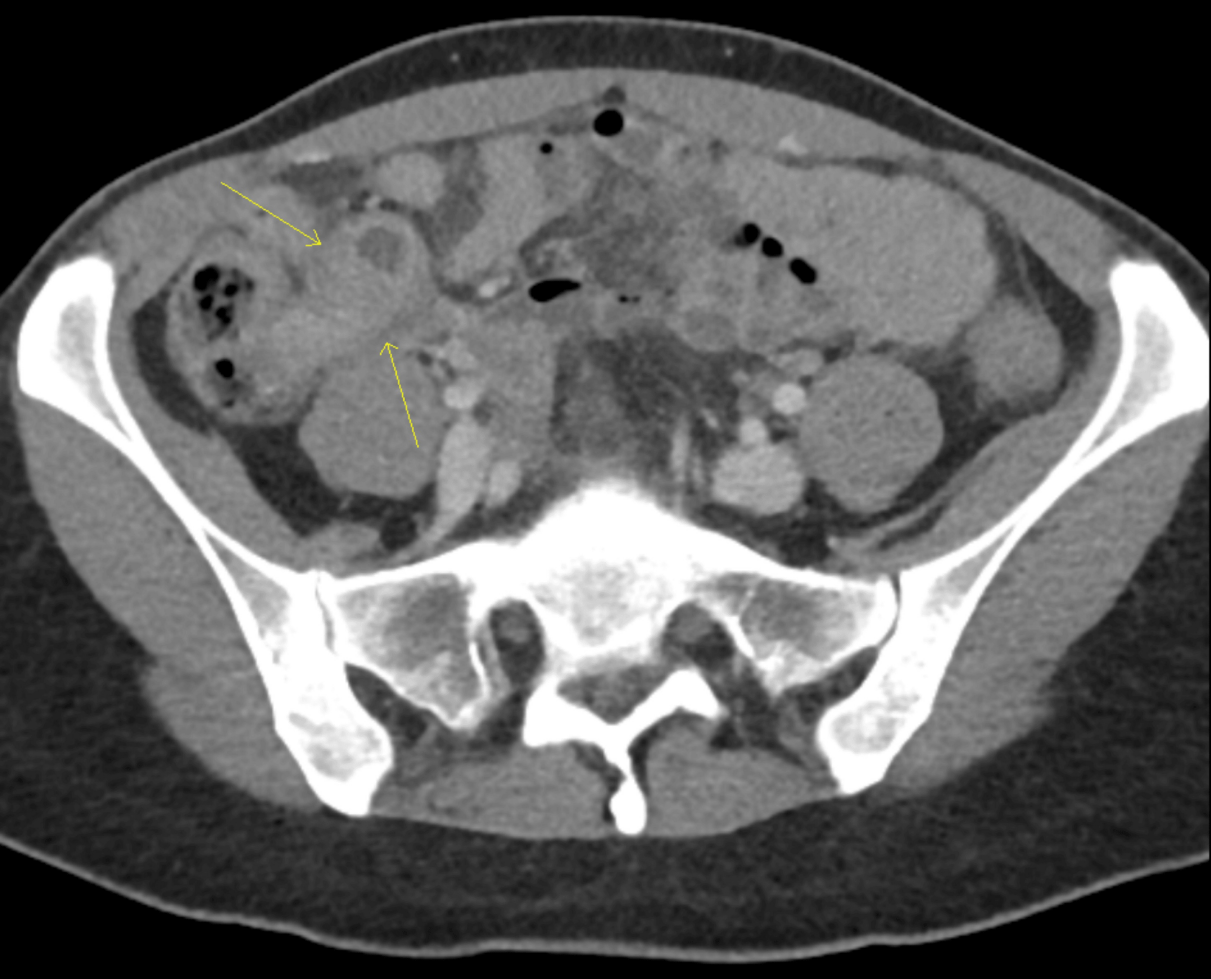
CT abdomen and pelvis with contrast showing a 3.7 x 1.8 x 3.0 cm phlegmon at base of cecum, consistent with acute complicated appendicitis (yellow arrows) CT: computed tomography

**Figure 2:**
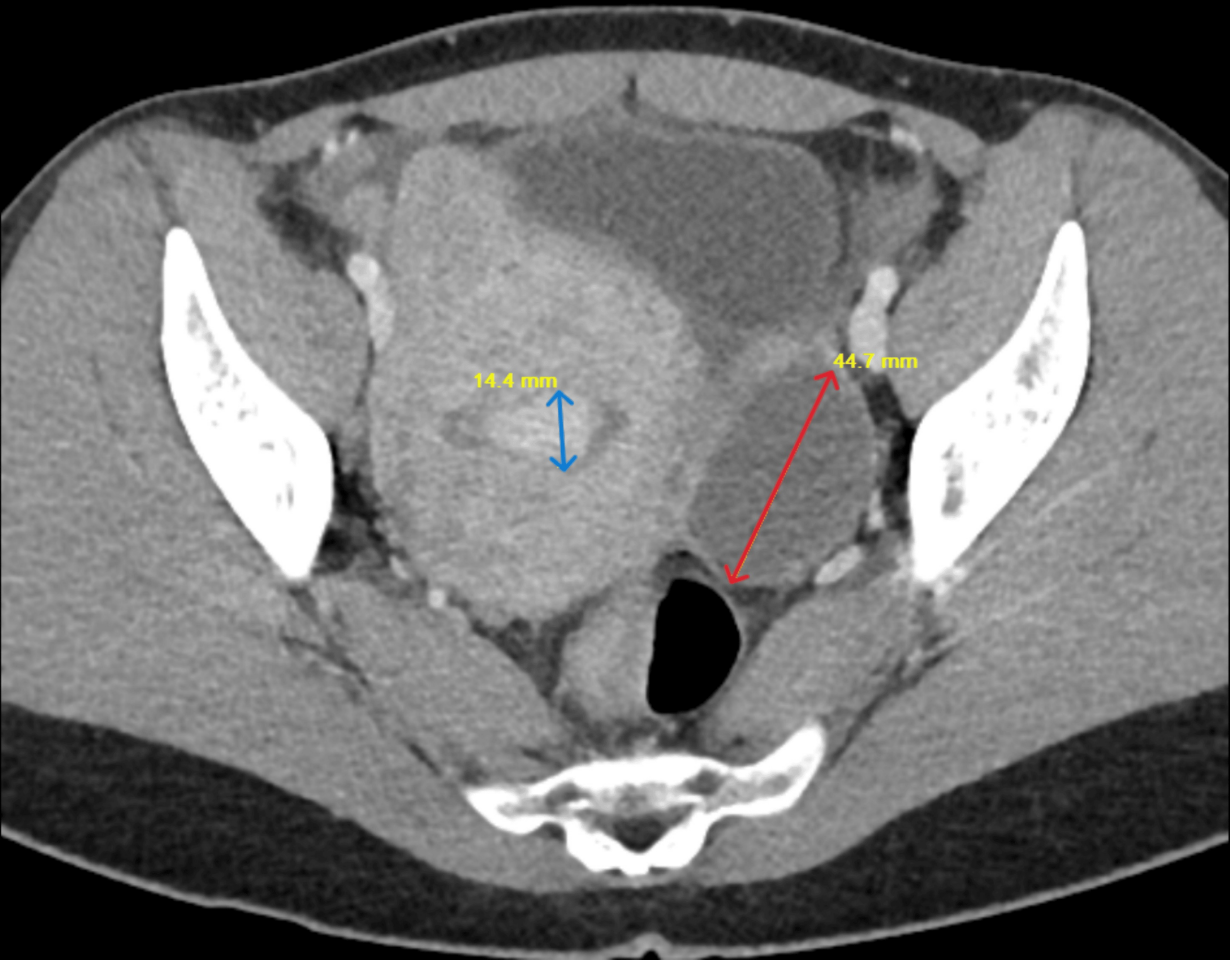
Repeat CT showing thickened endometrium (blue arrow: 14.4 mm) and endometrioma (red arrow: 44.7 mm) CT: computed tomography

Initial labs revealed anemia with a hemoglobin of 9.5 g/dL (reference range:12-16 g/dL), and leucocytosis of 16.4 × 10⁹/L (reference range: 4.0-11.0 × 10⁹/L). A contrast CT scan of the abdomen and pelvis showed acute appendicitis with phlegmon at the cecal base (Figure 1). CA19-9, CA-125, and CEA were sent, of which CA-125 was elevated: 92.7 U/mL (reference range: 0-35 U/mL). The patient was evaluated by colorectal surgery and admitted to the hospital for treatment of acute appendicitis with contained rupture. She was started on IV piperacillin-tazobactam, and an interval surgery was recommended. She reported mild improvement in symptoms. A repeat CT scan was done, which showed acute appendicitis with a phlegmon along with a small amount of free fluid, an enlarged heterogeneous uterus with a thickened endometrium, uterine fibroids, and a left ovarian cyst (Figure 2). Interventional Radiology drainage was discouraged due to concerns for a suspected malignant appendiceal mass, and the patient was treated with 14 days of antibiotics (IV piperacillin-tazobactam was switched to PO levofloxacin and metronidazole on discharge).

The patient underwent a colonoscopy; an appendiceal biopsy was done, which showed a serrated adenoma. Due to concerns for appendiceal malignancy, the patient underwent a laparoscopic appendectomy and a right hemicolectomy by a colorectal surgeon. The right colon was mobilized from medial to lateral in an avascular plane. High ligation of the ileocolic and right colic vessels was performed by dividing the mesentery at its base. The appendix was removed, and the colon was resected 10 cm proximally and distally from the lesion, along with excision of 22 lymph nodes. A side-to-side ileocolic anastomosis was performed with a tri-stapler. The specimens were retrieved and sent for histopathology. No complications, such as spillage, perforation, or rupture, were encountered.

**Figure 3:**
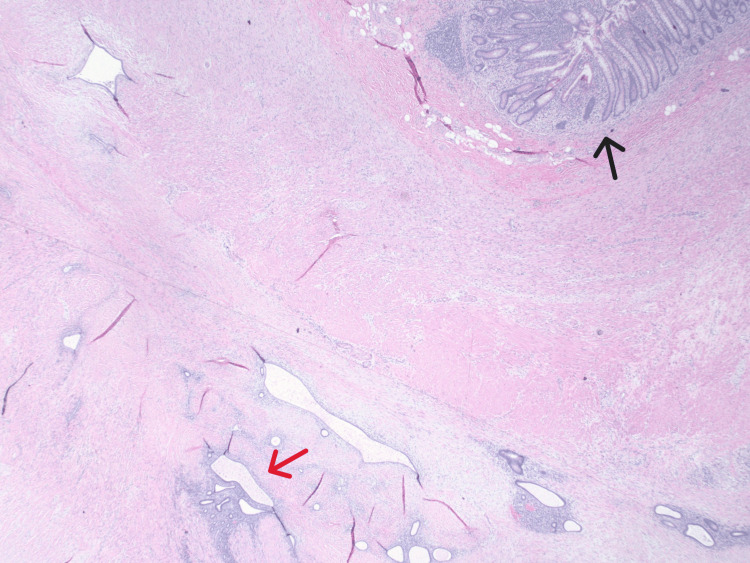
Histopathology showing endometrial tissue within the appendiceal wall (red arrow) adjacent to the appendiceal lumen (black arrow)

The final histopathology confirmed a 2.8 cm endometriotic lesion involving the wall of the appendix and surrounding tissue (Figure 3). The patient was referred to minimally invasive gynecologic surgery for the management of endometriosis and fibroids. Office hysteroscopy and endometrial biopsy revealed benign endocervical glandular and endocervical squamous tissue without evidence of atypical hyperplasia or malignancy. Pelvic MRI showed a left adnexal endometrioma measuring 3.6 x 3.2 cm, endometriosis along the posterior uterine fundus measuring 2.8 x 0.7 cm, multiple uterine leiomyomas, and adenomyosis. She expressed a desire to preserve some ovarian function, if clinically feasible, to allow for a natural transition to menopause. She subsequently underwent robotic-assisted hysterectomy, resection of endometriosis (sigmoid colon mesentery, bilateral pelvic sidewall, vaginal cuff, anterior abdominal wall), bilateral salpingectomy, left ovarian oophorectomy, right ovarian cystectomy for a 1 cm ovarian cyst, and cystoscopy with bilateral ureteral stents. Histopathologic examination confirmed endometriosis, with surgical findings meeting the criteria for stage IV disease.

The patient had an uncomplicated postoperative course and was discharged home. She confirmed the resolution of her pain during her post-op follow-up visit in the clinic. The final histopathology report confirmed the diagnosis of appendiceal endometrioma.

## Discussion

Endometriosis is one of the most common gynecological diseases. While it is commonly confined to the pelvis, there is literature supporting the presence of rare extra-pelvic endometriosis involving the GI tract, respiratory system, urinary tract, pleura, diaphragm, and pericardium [13,14]. The prevalence of endometriosis in the appendix is 1-3% in patients with endometriosis. [15]. Endometriosis of the appendix can have a similar presentation to pelvic endometriosis, including symptoms of dysmenorrhea, deep dyspareunia, and dyschezia, although melena, intussusception, and lower GI bleed have also been reported [16]. The current scientific literature provides various theories about the pathogenesis of endometriosis [17,18]. The common theories include retrograde menstruation, coelomic metaplasia, and benign metastasis (lymphatic, hematologic) [17]. Endometriosis has been accepted as a multifactorial disease process with multifactorial components ranging from genetic, immune, hormonal, and environmental components [4]. 

Sampson's theory proposes that endometrial tissue fragments are transplanted onto extrauterine organs during retrograde menstruation refluxes and subsequently proliferate into endometriosis [16,19]. Retrograde menstruation has become less supported within the endometriosis community as an independent process. This is partly because it does not explain diseases found outside of the pelvis, and also because most women experience retrograde menstruation without the development of endometriosis. This further supports the idea of a multifactorial disease process [6]. Coelomic metaplasia theory posits that normal peritoneal mesothelium undergoes metaplastic transformation into endometrial tissue. In contrast, the induction theory suggests that undifferentiated mesothelial cells differentiate into endometrial cells due to an endogenous inductive stimulus, which may be hormonal or immunological. Additionally, the presence of distant endometriotic lesions supports the lymphatic and hematologic dissemination of endometrial cells, thus favoring the "benign metastasis" theory [7,17]. 

MRI should be used for localization, developing differential diagnoses, and ruling out other conditions such as masses, hernias, and lipomas. However, preoperative diagnosis remains challenging, as there are no reported radiological features that describe appendiceal endometriosis [12]. There are endometriosis centers that may have the expertise to offer MRI and ultrasound protocols for identifying endometriosis in the pelvis or involving the distal colon before surgery; however, there is no evidence-based criteria for identifying appendiceal disease.

Treatment of extrapelvic endometriosis can start with medication intervention; however, surgical excision is typically needed as medical therapies are often ineffective in these cases [1]. Management of endometriosis of the appendix is often determined at the time of surgery due to diagnostic limitations. Within the gynecological community, appendectomy is supported if a gross abnormality is found and can still be considered in the case of persistent RLQ pain alone [20]. Appendectomy for an appendix without gross abnormality at the time of surgery for endometriosis lacks supportive prospective data. Retrospective data strongly support considering this intervention as it does not increase operative morbidity and carries potential preventative, diagnostic, and even pain treatment value, even when appearing normal [15,21-22]. Current general surgery literature advocates for the treatment of appendiceal endometriosis through appendectomy, right hemicolectomy, or ileocecectomy [8]. The extent of surgical intervention should be based on the severity of endometriosis involvement. It is crucial to perform a meticulous examination of the abdominopelvic cavity to evaluate the extent of endometriosis involvement and to consider a multidisciplinary team approach if other organs are affected [8].

Our patient exhibited signs and symptoms of acute appendicitis with an appendiceal mass, which was confirmed to be endometriosis of the appendix on pathology. The patient had not undergone any prior investigation for or received a diagnosis of endometriosis, but had stage IV endometriosis on pelvic exploration. The patient underwent surgery with a good surgical outcome. We encourage physicians to consider endometriosis in the differential diagnosis when encountering women of reproductive age presenting with acute RLQ pain.

## Conclusions

Extragonadal endometrioma is a rare manifestation of endometriosis. This report highlights the importance of considering extragonadal endometriosis in reproductive-age women presenting with RLQ pain, acute appendicitis, and appendiceal mass. It illustrates how an appendiceal endometrioma mimics acute appendicitis both clinically and radiologically. Although intraoperative and histopathological evaluation is needed to establish a definitive diagnosis, medical professionals in the emergency department, gynecologists, and general surgeons must maintain a heightened level of suspicion for endometriosis, especially in female patients presenting with lower abdominal pain, to prevent misdiagnosis and achieve optimal patient outcomes.
